# Juno spacecraft gravity measurements provide evidence for normal modes of Jupiter

**DOI:** 10.1038/s41467-022-32299-9

**Published:** 2022-08-30

**Authors:** Daniele Durante, Tristan Guillot, Luciano Iess, David J. Stevenson, Christopher R. Mankovich, Steve Markham, Eli Galanti, Yohai Kaspi, Marco Zannoni, Luis Gomez Casajus, Giacomo Lari, Marzia Parisi, Dustin R. Buccino, Ryan S. Park, Scott J. Bolton

**Affiliations:** 1 Department of Mechanical and Aerospace Engineering, Sapienza University of Rome, Rome, Italy; 2 Observatoire de la Côte d’Azur, Université Côte d’Azur, CNRS, Nice, France; 3 Division of Geological and Planetary Sciences, California Institute of Technology, Pasadena, CA USA; 4 Department of Earth and Planetary Sciences, Weizmann Institute of Science, Rehovot, Israel; 5 Department of Industrial Engineering, University of Bologna, Forlì, Italy; 6 Department of Mathematics, University of Pisa, Pisa, Italy; 7 Jet Propulsion Laboratory, California Institute of Technology, Pasadena, CA USA; 8 Southwest Research Institute, San Antonio, TX USA

**Keywords:** Seismology, Giant planets

## Abstract

The Juno spacecraft has been collecting data to shed light on the planet’s origin and characterize its interior structure. The onboard gravity science experiment based on X-band and Ka-band dual-frequency Doppler tracking precisely measured Jupiter’s zonal gravitational field. Here, we analyze 22 Juno’s gravity passes to investigate the gravity field. Our analysis provides evidence of new gravity field features, which perturb its otherwise axially symmetric structure with a time-variable component. We show that normal modes of the planet could explain the anomalous signatures present in the Doppler data better than other alternative explanations, such as localized density anomalies and non-axisymmetric components of the static gravity field. We explain Juno data by p-modes having an amplitude spectrum with a peak radial velocity of 10–50 cm/s at 900–1200 μHz (compatible with ground-based observations) and provide upper bounds on lower frequency f-modes (radial velocity smaller than 1 cm/s). The new Juno results could open the possibility of exploring the interior structure of the gas giants through measurements of the time-variable gravity or with onboard instrumentation devoted to the observation of normal modes, which could drive spacecraft operations of future missions.

## Abstract (web summary)

Juno spacecraft experienced unknown accelerations near the closest approach to Jupiter. Here, the authors show that Jupiter’s axially symmetric, north-south asymmetric gravity field measured by Juno is perturbed by a time-variable component, associated to internal oscillations.

## Introduction

The Juno spacecraft has been orbiting Jupiter in a highly eccentric, 53.5-day orbit since July 2016. After the 33rd perijove passage in April 2021 (labeled PJ33), the mission ended its prime mission and entered its extended mission. In the prime mission, the hemispherically symmetric part of the observed gravity field has been used to infer the possible existence of a dilute core^1^. The north-south asymmetric part of Jupiter’s gravity field was also determined^2^, with an amplitude and pattern implying that the zonal winds extend to a few thousand kilometers depth^3^. These analyses assumed a zonal gravity field (i.e., no longitudinal dependence).

**Fig. 1:**
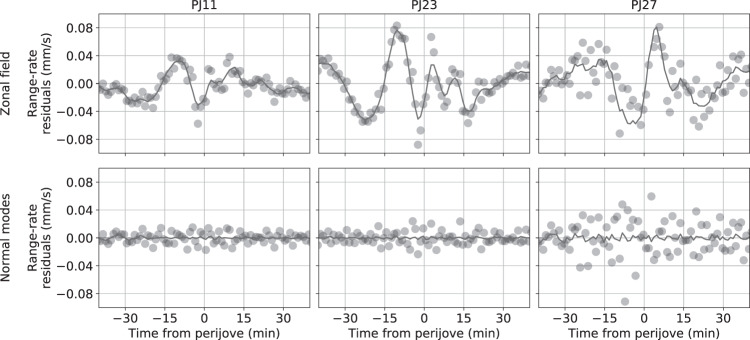
Juno two-way range-rate (Doppler) residuals in mm/s for selected passes. Different dynamical models are compared: static zonal gravity field (first row) or including normal modes (second row). The light black line is a moving average of the residuals, to highlight signatures near perijove if a zonal field is assumed. See Supplementary Figure 1 for Doppler residuals in all Juno perijove passes and different dynamical models.

Recently, the analysis of Doppler data collected by Juno up until the middle of its prime mission^4^ reported the need to include additional accelerations at the level of 2–5 × 10^−8^ m/s^2^ to fit the data. If Jupiter’s gravity representation is limited to zonal harmonics the data shows signatures at up 0.1 mm/s over timescales of 10–15 min (see Fig. 1, top panels), to be compared with the accuracy of ~0.01 mm/s at 60 s provided by Juno’s radio instrumentation. Similar unexplained accelerations, with 20 times larger amplitude, have been observed on the Cassini spacecraft^5^ during the Grand Final orbits about Saturn^6^. Empirical accelerations can be used to describe these additional spacecraft motions, thus avoiding a specific physical explanation. Although they are assumed to be uncorrelated in time, their amplitude peaks near the pericenters. Plausible phenomena are density anomalies embedded in the differentially rotating outer shell (hence time-dependent in Jupiter’s System III reference frame^7^), non-zonal density anomalies at depth (necessarily dynamic but almost time-independent on the mission timescale), or internal oscillations, i.e., normal modes (the focus of this study).

An example of the first phenomenon is the recently detected gravity anomaly associated with the Great Red Spot (GRS)^8^. When Juno flew over the anticyclonic storm, an acceleration comparable to the unexplained signal have been observed (3 × 10^−8^ m/s^2^).

Since Jupiter’s normal modes can potentially displace large internal masses, the gravity field can show a time-variable component. This is described by the planetary seismology discipline. Currently, the best proposed evidence of Jupiter’s p-modes (oscillations whose restoring force is pressure) has been provided by Gaulme et al.^9^, who analyzed Earth-based, low spatial resolution radial velocity maps of Jupiter’s surface and found an excess power at frequencies between 800 and 1500 μHz. Lower frequency fundamental (f-)modes (those having radial order *n* = 0, thus penetrating deeper in the planet) could have not been detected due to limitations in the ground instrumentation. At Saturn, several ring observations provided different kind of insights into its oscillation spectrum. Stellar occultations measurements in Saturn’s rings revealed more than 30 f-modes inside the planet which affect the rings^10–13^. Ring seismology proved to be successful in constraining Saturn’s interior^14,15^ due to the accurate measurements of f-modes frequencies, having amplitudes of the order of 60 cm and velocities of 0.06 cm/s^14^. Additionally, the analysis of residual accelerations of the Cassini spacecraft during the Grand Finale orbits^16^ provided evidence for p-modes in Saturn’s oscillation spectrum. Remarkably, Cassini’s gravity data did not show evidence for f-modes, even though they have a larger gravity signal for a given amplitude of motion (the radial eigenfunction does not have any node). The gravity and ring data are mutually consistent because the rings are exquisitely sensitive to f-modes and completely insensitive to p-modes. The evidence for normal modes at Saturn motivates a similar search in Juno’s data. Previous numerical simulations^17^, based on the amplitude spectrum of Gaulme et al.^9^, predicted that Jupiter’s normal modes should be observable in Juno’s Doppler data.

In this work, we focus on internal oscillations, or normal modes, as a possible cause of the unexplained accelerations and show that temporal variations in the gravity field of Jupiter caused by normal modes are compatible with Juno’s Doppler data. The best-fit amplitude spectrum has a peak radial velocity of 10–50 cm/s at 900–1200 μHz (p-modes regime), while lower frequency, f-modes has to be smaller than 1 cm/s. In addition, we show that normal modes provide a better fit of Juno’s data with respect to other physical phenomena. The analysis includes gravity-dedicated passes up to PJ33, for a total of 22 passes (see Methods, subsection data set).

## Results

The unambiguous identification of single normal modes from Juno Doppler data is not possible if the observed accelerations arise from multiple modes. In addition, Juno’s orbit is far from ideal to map a time-varying gravity field, because, as a consequence of the large eccentricity, relevant observations are concentrated to a few hours around perijove, repeating every 53.5 days over different Jovian longitudes. Since typical normal modes periods range from 10 min to two hours, the short observation window (6–8 h) limits the observability of time-varying phenomena.

We, therefore, focus our analysis on the identification of spectral amplitudes compatible with the observed signatures in Juno’s Doppler data. After having selected an empirical model describing those amplitudes, we carry out the analysis of Juno’s Doppler data by varying the model parameters and identifying the region of the parameter space that shows maximum compatibility with the observations, according to the Akaike information criterion (AIC)^18^.

### Jupiter’s normal modes model

To compute the oscillation spectrum, we follow the approach detailed in Durante et al.^17^. Since for the purpose of this work it is not necessary to construct accurate eigenfrequencies, we use a non-rotating polytropic structure of index 1 for Jupiter’s interior, and compute the eigenfrequencies ${\omega }_{{lmn}}$ and radial eigenfunctions ${\xi }_{{lmn}}\left({r}^{{\prime} }\right)$ (with *l*, *m*, *n* being, respectively, the degree, order, and radial order of a given mode) with GYRE^19^. The density perturbation is proportional to the radial eigenfunction and density gradient, oscillating at a given angular frequency:

1 $${\widetilde{\Delta \rho }}_{{lmn}}\left({r}^{{\prime} },\,{\theta }^{{\prime} },\,{\varphi }^{{\prime} }\right)={A}_{{lmn}}\frac{\partial \rho \left(r^{\prime} \right)}{\partial {r}^{{\prime} }}\,{\xi }_{{lmn}}\left({r}^{{\prime} }\right)\,{Y}_{{lm}}\left({\theta }^{{\prime} },\,{\varphi }^{{\prime} }\right)$$

2 $${\Delta \rho }_{{lmn}}({r}^{{\prime} },\,{\theta }^{{\prime} },\,{\varphi }^{{\prime} },\,t)={\widetilde{\Delta \rho }}_{{lmn}}\left({r}^{{\prime} },\,{\theta }^{{\prime} },\,{\varphi }^{{\prime} }\right){{\cos }}\left({\omega }_{{lmn}}t+{\phi }_{{lmn}}\right)$$

with ${A}_{{lmn}}$ being the surface peak amplitude, ${Y}_{{lm}}$ the spherical harmonic function, and ${\phi }_{{lmn}}$ an unknown phase. We ignore lateral derivatives of the density profile and the lateral displacement of the fluid. To compute the perturbation to the harmonic coefficients, we integrate over the volume of the planet:

3 $${\widetilde{C}}_{{lmn}}=\frac{{\int }_{V}{{r}^{{\prime} }}^{l}{Y}_{{lm}}\left({\theta }^{{\prime} },\,{\varphi }^{{\prime} }\right){\widetilde{\Delta \rho }}_{{lmn}}\left({r}^{{\prime} },{\theta }^{{\prime} },\,{\varphi }^{{\prime} }\right)d{V}^{{\prime} }}{\left(2l+1\right)M{R}^{l}}$$

with *M* and *R* being, respectively, the mass and equatorial radius of Jupiter. The total contribution to a given coefficient is:

4 $$\Delta {C}_{{lm}}(t)={\sum }_{n}{\widetilde{C}}_{{lmn}}{{\cos }}\left({\omega }_{{lmn}}t+{\phi }_{{lmn}}\right)$$

**Fig. 2:**
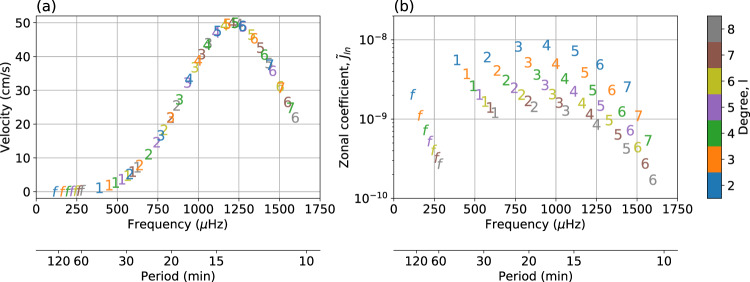
Normal modes model. Radial velocity profile (**a**) and corresponding amplitude of normalized spherical harmonic coefficients (**b**). The model is depicted for ${{{{\mathcalligra{v}}}}}_{{{{{{{\rm{max}}}}}}}}$ = 50 cm/s, zero ${{{{\mathcalligra{v}}}}}_{{{{{{{\rm{min}}}}}}}}$, ${f}_{{{{{{{{\rm{peak}}}}}}}}}$ = 1210 μHz, and *σ*_*f*_ = 300 μHz. The letters *f* and the numbers indicate, respectively, f-modes and the radial order of p-modes, up to *n* = 7.

Although the background density profile and eigenfunctions of a polytrope differ from those of equation of state-based models, we find that the differences in ${\widetilde{C}}_{{lmn}}$, for a given surface amplitude, are limited to <20% for modes with frequency less than ~1000 μHz, and <50% for modes with frequency up to ~1350 μHz. These differences are well within the intrinsic scatter in the coefficient spectrum as a function of *l* or *n* over the relevant frequency range (see Fig. 2b), justifying the choice of modeling the interior with a polytrope. In our analysis, we limit the range of normal modes to zonal coefficients ${\widetilde{J}}_{{ln}}$ (*m* = 0) to avoid excessive overparameterization of Juno’s dynamical model. Moreover, the polar orbit and the large eccentricity of Juno lead to a rapid variation in the gravity acceleration near the perijove and thus has an ambiguous interpretation, preventing good distinction between high-order zonal and tesseral harmonics. Nevertheless, we verified that results are robust with the inclusion of tesseral coefficients.

Finally, to compute the amplitudes for a given model, we assume that the radial velocity associated with each mode depends only on its oscillation frequency, and not on its degree or radial order, as suggested by solar p-modes observations^20^. We model the amplitude ${A}_{{lmn}}$ of each mode by assuming a frequency-dependent Gaussian profile (see Fig. 2a) for the surface radial velocity, ${{{{\mathcalligra{v}}}}}$, on top of a possible background noise:

5 $${{{\mathcalligra{v}}}}\left(f\right)={{{{\mathcalligra{v}}}}}_{{{{{{{{\rm{min }}}}}}}}}+\left({{{\mathcalligra{v}}}}_{{{{{{{{\rm{max }}}}}}}}}-{{{\mathcalligra{v}}}}_{{{{{{{{\rm{min }}}}}}}}}\right){{{{{{{\rm{exp }}}}}}}}\left[-{\frac{1}{2}\left(\frac{f-{f}_{{{{{{{{\rm{peak}}}}}}}}}}{{\sigma }_{f}}\right)}^{2}\right]$$

6 $${A}_{{lmn}}=\frac{{{{\mathcalligra{v}}}}\left({f}_{{lmn}}\right)}{2\,\pi \,{f}_{{lmn}}}$$

Our radial velocity-to-frequency model entails four parameters: the minimum velocity, ${{{{\mathcalligra{v}}}}}_{{{{{{{\rm{min}}}}}}}}$, the maximum velocity, ${{{{\mathcalligra{v}}}}}_{{{{{{{{\rm{max}}}}}}}}}$, at peak frequency, ${f}_{{{{{{{{\rm{peak}}}}}}}}}$, and the spread of the Gaussian, *σ*_*f*_. The choice of a Gaussian profile is justified since it well approximates a resonance-like phenomenon: if we suppose the acoustic modes are stochastically excited and intrinsically damped by convection and turbulent viscosity (or other phenomena), they behave like a forced and damped oscillator (e.g., for the Sun spectrum see Fröhlich et al.^21^ and García et al.^22^). The parameters of the model that best fits the first of the two frequency regions where excess power was found by Gaulme et al.^9^ are: ${{{{\mathcalligra{v}}}}}_{{{{{{{{\rm{max }}}}}}}}}$ = 49 cm/s, ${f}_{{{{{{{{\rm{peak}}}}}}}}}$ = 1210 μHz, and *σ*_*f*_ = 300 μHz. The ${{{{\mathcalligra{v}}}}}_{{{{{{{{\rm{min }}}}}}}}}$ parameter is undetermined since the noise floor was large, especially at the lower frequencies. Although ${{{{\mathcalligra{v}}}}}_{{{{{{{{\rm{min }}}}}}}}}$ overparameterize the measured spectrum, it could catch any excess power in lower frequency f-modes. According to simulations presented in Durante et al.^17^, the scenario with ${{{{\mathcalligra{v}}}}}_{{{{{{{{\rm{min }}}}}}}}}$ = 30 cm/s can be ruled out because it would produce spacecraft accelerations that are too large.

### Multiarc least square estimation filter

Juno’s Doppler data have been fitted using a multi‐arc least square estimation filter. Data acquired by Juno during a pericenter pass forms an observation arc. For each arc, we solve for a set of local parameters: Juno’s initial position and velocity, the velocity change during Earth repointing turns (they may occur after the perijove pass, before the orbit trim maneuver), and normal modes amplitudes. Global parameters (common to all arcs) are Jupiter’s gravitational parameter (GM), zonal harmonic coefficients to degree 30, non‐zonal coefficients of degree-2, tidal Love numbers up to degree and order 4, Jupiter’s spin axis initial position, and polar moment of inertia factor, the mass of the GRS dipole, and a scale factor for the solar radiation pressure. Except for normal modes amplitudes, no a priori uncertainty constraint has been imposed on any parameter. The reader should refer to the dedicated Method’s section for more details on Juno’s dynamical model.

Concerning normal modes, it is impossible to fit the data by assuming phase coherence of the oscillations across all observation arcs, given the large gap between the pericenter passes and the uncertainty in the frequency of the modes. For a given solution on our search grid, we, therefore, estimate the amplitude and phase (the amplitudes of a cosine and sine term) of each mode as local parameters (pertaining to a single observation arc). Furthermore, we include only those modes having: (1) a spherical harmonic degree up to 8, since higher order harmonics are mutually highly correlated due to Juno’s eccentric orbit and would not be accessible, and (2) a period larger than 10 min (that is, a radial order up to 7 at most) since Juno’s 60 s Doppler data would not provide sufficiently dense measurements to distinguish higher-frequency modes. Our choice is driven by two factors: the noise in the Doppler data (higher at shorter integration times) and the need to avoid overparameterization of Juno’s dynamical model. The signal coming from the neglected (i.e., truncated) modes is, per unit amplitude, smaller than the signal from lower frequency, lower order modes. Then, we estimate a mode only if the amplitude of the spherical harmonic coefficient is larger than ~10^−11^: smaller values would not be large enough to affect Juno’s orbit in any detectable way (e.g., with the current data set, Juno’s sensitivity to static *J*_*2*_ is ~2 × 10^−10^). For this reason, the number of modes included in each solution can vary according to model parameters. The maximum number of modes is 56, which is reached for ${{{{\mathcalligra{v}}}}}_{{min }}$, ${{{{\mathcalligra{v}}}}}_{{max }}$ larger than ~3 mm/s. For each mode, we start from an a priori value of zero (since we do not know the initial phase) and set an a priori uncertainty derived from the model parameters in the search grid. That is, the filter can estimate the amplitude and phase of each mode under the (soft) constrain given by model of normal modes amplitudes. Since we do not impose the amplitude of the modes but their a priori uncertainty, the filter may occasionally estimate amplitudes that exceed the model constraints. In this case, the solution shall be rejected (i.e., penalized, see next paragraph), because the model amplitudes given by that set of parameters are not compatible with the data.

### Model selection: AIC

We performed the task of model selection using the AIC^18^. This well-established statistical criterion is based on the use of entropy as a measure of information and provides a quantitative value of the information loss when a given data fitting model is selected. It selects models using their likelihood *L* in fitting the Doppler data while favoring low complexity models, expressed by the associated regression-effective degree of freedom *k*:

7 $${AIC}=2\,k-2\,{{\log }}\,L$$

AIC consists of a trade-off between the goodness of fit and the simplicity of the model itself. Its minimization requires the maximization of the (log-) likelihood function of the model to the data, while keeping the solution’s degrees of freedom to minimum (low value of *k*). See dedicated subsection in Methods for how to compute the number of degrees of freedom of a given dynamical solution. In the case of a least square estimation filter, by substituting the corresponding likelihood function, the AIC can be computed as:

8 $${AIC}=2\,k+{n}_{{{{{{{{\rm{obs}}}}}}}}}{{\log }}\,{RS}{S}_{{{{{{{{\rm{obs}}}}}}}}}+C$$

With *n*_obs_ being the number of Doppler data points, *RSS*_obs_ their root-sum-square, normalized with the noise standard deviation, and *C* a constant which depends only on the number of data points (which is unchanged in the different solutions we aim to compare).

Given the possibility of incurring into a solution with a large difference between estimated and model amplitudes for the normal modes, the likelihood is constructed as the product of the data-driven and amplitude-based likelihoods. The additional, amplitude-based, likelihood term is:

9 $${L}_{m}=\frac{1}{{\left(2\pi {\hat{\sigma }}^{2}\right)}^{{n}_{{{{{{{{\rm{modes}}}}}}}}}/2}}{{\exp }}\left(-\frac{1}{2}{RS}{S}_{{{{{{{{\rm{modes}}}}}}}}}\right)$$

here *n*_modes_ is the number of modes estimated (for all the arcs), *RSS*_modes_ the root-sum-square of the difference between the model and estimated amplitudes of the modes, normalized to the uncertainty of the mode amplitude, and $\hat{\sigma}$ = *RSS*_modes_/*n*_modes_ is the maximum likelihood estimate of the variance of a model’s residuals distribution. This term becomes important when the data are not compatible with the amplitude of the modes we try to impose. The final AIC function reads:

10 $${AIC}=2\,k+{n}_{{{{{{{{\rm{obs}}}}}}}}}{{\log }}\,{RS}{S}_{{{{{{{{\rm{obs}}}}}}}}}+{n}_{{{{{{{{\rm{modes}}}}}}}}}{{\log }}\left(2\pi \frac{{RS}{S}_{{{{{{{{\rm{modes}}}}}}}}}}{{n}_{{{{{{{{\rm{modes}}}}}}}}}}\right)+\frac{2}{{n}_{{{{{{{{\rm{modes}}}}}}}}}}+C$$

Since only differences in AIC among different solutions are important, we select the constant *C* to have AIC equal to zero for the best solution found in the analysis. Gravity solutions with a small AIC value are selected as the best models. Generally, solutions having ΔAIC larger than ~500 still show signature in the Doppler data of a few passes.

### Looking for normal modes

We run more than three thousand simulations, exploring the parameter space given by the four parameters of our model for the amplitudes of normal modes, on a suitable search grid. The range of variability of each parameter is: ${{{{\mathcalligra{v}}}}}_{{{{{{{{\rm{min }}}}}}}}}$ = 1 mm/s–10 m/s, ${{{{\mathcalligra{v}}}}}_{{{{{{{{\rm{max }}}}}}}}}$ = 1 mm/s–10 m/s, ${f}_{{{{{{{{\rm{peak}}}}}}}}}$ = 600–1500 μHz, *σ*_*f*_ = 250–350 μHz, with the limitation ${{{{\mathcalligra{v}}}}}_{{{{{{{{\rm{max }}}}}}}}}$ > ${{{{\mathcalligra{v}}}}}_{{{{{{{{\rm{min }}}}}}}}}$, to avoid having a downward pointing Gaussian profile.

The range-rate (Doppler) residuals do not show leftover signatures when suitable normal modes are included in Juno’s dynamical model (see selected passes on Fig. 1, bottom panels, or Supplementary Figure 1, for a more comprehensive comparison).

**Fig. 3:**
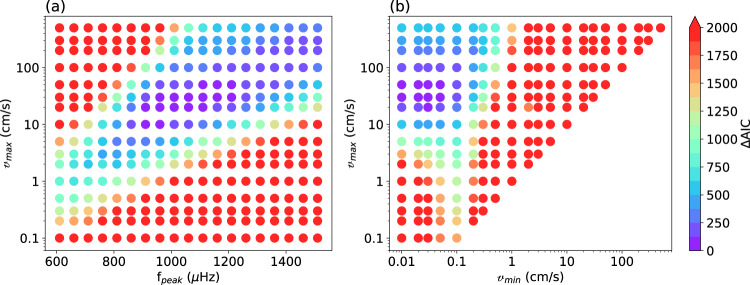
Results for two slices of the full parameter space. **a** ΔAIC value as a function of ${{{{\mathcalligra{v}}}}}_{{{{{{{{\rm{max }}}}}}}}}$ and *f*_peak_, with ${{{\mathcalligra{v}}}}_{{{{{{{{\rm{min }}}}}}}}}$ = 0. **b** ΔAIC value as a function of ${{{{\mathcalligra{v}}}}}_{{{{{{{{\rm{max }}}}}}}}}$ and ${{{{\mathcalligra{v}}}}}_{{{{{{{{\rm{min }}}}}}}}}$, with *f*_*peak*_ = 1200 μHz. Both slices have *σ*_*f*_ = 300 μHz. Each circle is a different solution.

Figure 3 show two slices of the full parameter space. Panel a reports the ΔAIC value for solutions obtained by varying ${{{{\mathcalligra{v}}}}}_{{{{{{{{\rm{max }}}}}}}}}$ and ${f}_{{{{{{{{\rm{peak}}}}}}}}}$, with *σ*_*f*_ = 300 μHz and ${{{{\mathcalligra{v}}}}}_{{{{{{{{\rm{min }}}}}}}}}$ = 0. The best solutions (having minimum ΔAIC value) are those in the region having ${{{{\mathcalligra{v}}}}}_{{{{{{{{\rm{max }}}}}}}}}$ = 10–50 cm/s and ${f}_{{{{{{{{\rm{peak}}}}}}}}}$ = 900–1200 μHz, compatible with ground-based observations by Gaulme et al.^9^.

Figure 3b reports the ΔAIC value for solutions obtained by varying ${{{{\mathcalligra{v}}}}}_{{{{{{{\rm{max}}}}}}}}$ and ${{{{\mathcalligra{v}}}}}_{{{{{{{\rm{min}}}}}}}}$, with ${f}_{{{{{{{{\rm{peak}}}}}}}}}$ = 1200 μHz and *σ*_*f*_ = 300 μHz. The solutions having minimum ΔAIC value are those having ${{{{\mathcalligra{v}}}}}_{{{{{{{\rm{max}}}}}}}}$ = 10–50 cm/s and ${{{{\mathcalligra{v}}}}}_{{{{{{{\rm{min}}}}}}}}$ smaller than 1 mm/s, effectively contributing very little to the final frequency profile. The ${{{{\mathcalligra{v}}}}}_{{{{{{{\rm{max}}}}}}}}$ parameter is compatible with Gaulme et al.^9^ observations, while the small values of ${{{{\mathcalligra{v}}}}}_{{{{{{{\rm{min}}}}}}}}$ suggest Jupiter’s f-modes have small amplitudes.

**Fig. 4:**
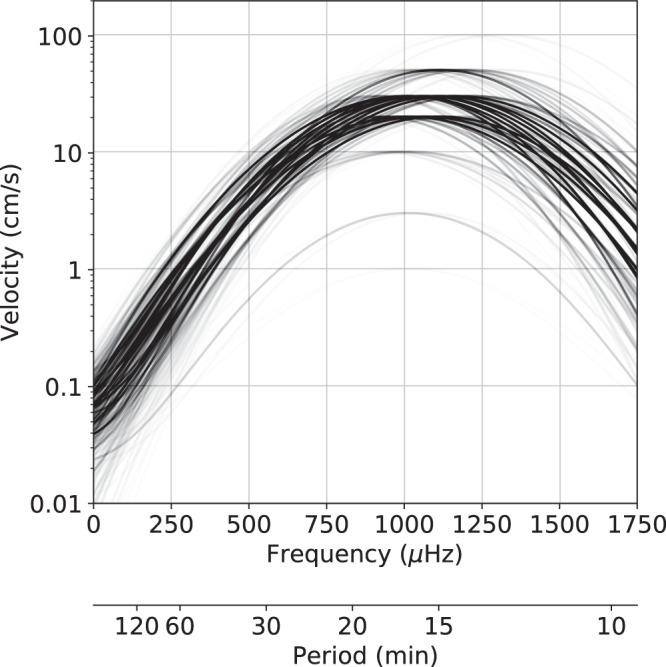
Recovered radial velocity profile as a function of frequency. Each line corresponds to a different model in the search grid. The opacity is proportional to Akaike weights: darker lines are for more likely models (decreasing ΔAIC values), conversely, lighter lines are for gradually less probable models (increasing ΔAIC values). The spread of the lines provides an indication of the uncertainty of the recovered amplitude spectrum.

The general result is presented in Fig. 4. It reports the radial velocity profile for the best models found according to the ΔAIC value, on the full search grid. The opacity of each line is proportional to the Akaike weights, that is, proportional to exp(-ΔAIC/2). The most probable solutions have a peak frequency in the order of 1000–1200 μHz, with a width of ~300 μHz. The maximum radial velocity ${{{{\mathcalligra{v}}}}}_{{{{{{{\rm{max}}}}}}}}$ is in the range 10–50 cm/s (or 15–80 m in surface displacement), with ${{{{\mathcalligra{v}}}}}_{{{{{{{\rm{min}}}}}}}}$ < 1 mm/s, and f-modes amplitudes of ~1–10 mm/s (or 2–6 m), indicating a strong likelihood for large p-modes and small f-modes. Although a comparison between normal modes on Jupiter and the Sun is not justified at this point, we note that the ratio between amplitudes of p-modes and f-modes is the same for the two bodies, about 20–100, in terms of radial velocity (e.g., Fröhlich et al.^21^). It is unclear whether a similar ratio may point to similar excitation mechanisms.

In addition, we test energy equipartition by simplifying the frequency profile and assuming a flat velocity profile as a function of the frequency (${{{{{{{\mathcalligra{v}}}}}}}}\left(f\right)={{{{{{{{\mathcalligra{v}}}}}}}}}_{{{{{{{{\rm{uniform}}}}}}}}}$). In such a scenario, f-modes largely dominates the gravity perturbation for two main reasons: (1) they penetrate deeper into the planet and displace more mass than p-modes for a given surface amplitude, and (2) at a given velocity, the amplitude of lower frequency modes is larger than the higher-frequency ones. That is, the spectrum of gravity coefficients is dominated by f-modes, with p-modes producing smaller perturbations, especially for higher degree, higher radial order modes.

**Fig. 5:**
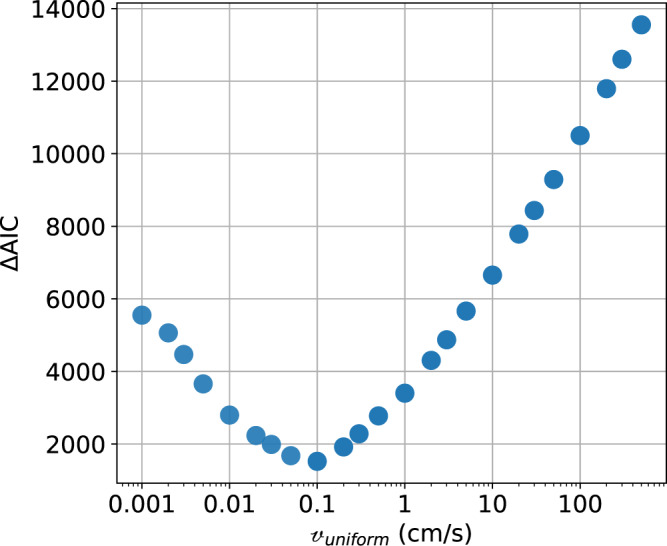
ΔAIC values as a function of ${{{{\mathcalligra{v}}}}}_{{{{{{{\rm{uniform}}}}}}}}$, for a flat velocity profile with ${{{\mathcalligra{v}}}}(f)={{{{\mathcalligra{v}}}}}_{{{{{{{{\rm{uniform}}}}}}}}}$. The minimum ΔAIC corresponds to a solution that does not fit Juno Doppler data: such a model does not represent adequately Juno’s data.

The result of the analysis indicates that the assumption of energy equipartition (or, alternatively, a model with dominant f-modes) does not fit the data, i.e., produces solutions which are not as good as those invoking higher-frequency modes. Figure 5 reports the ΔAIC as a function of the free parameter ${{{{{{{{\mathcalligra{v}}}}}}}}}_{{{{{{{\rm{uniform}}}}}}}}$. The minimum ΔAIC value of 1520 (at ${{{{{{{{\mathcalligra{v}}}}}}}}}_{{{{{{{{\rm{uniform}}}}}}}}}$ = 1 mm/s) is much larger than the ΔAIC of the best model found with dominating p-modes (which we recall have ΔAIC = 0). For models with large ${{{{{{{{\mathcalligra{v}}}}}}}}}_{{{{{{{{\rm{uniform}}}}}}}}}$, the estimated amplitudes of the modes largely deviate from those of the model, and the corresponding term of the likelihood function (Eq. 9) strongly penalizes these solutions. Fundamental modes become systematically lower than the model’s amplitudes: another indication that some power on p-modes is required. That is, energy equipartition (i.e., a model with ${{{{{{{{\mathcalligra{v}}}}}}}}\left(f\right){{{{{{{\mathcalligra{=}}}}}}}}{{{{{{{\mathcalligra{v}}}}}}}}}_{{{{{{{{\rm{uniform}}}}}}}}}$) is not adequate to represent Juno’s data.

## Discussion

The goal of our work is to assess whether the unexplained accelerations of the Juno spacecraft near the closest approach to Jupiter could be attributed to normal modes inside Jupiter. With this assumption, we report the amplitude spectrum derived with the Juno gravity measurements. Alternative explanations, such as a static tesseral field or localized density anomalies have been analyzed and ruled out (see Supplementary Information). The unexplained signal cannot be explained by a small set of lowest order tesseral harmonics (see Methods, subsection static tesseral field) thus excluding a hypothetical high-viscosity small core source. Non-zonal, spectrally rich but deep-seated gravity anomalies imply laterally varying density at depth, which can arise from thermal anomalies, flows (i.e., variations in dynamical pressure) or even large magnetic fields. At present, there are no reliable estimates of such features from existing models of convection or field generation in a fluid body because the correct parameters (especially viscosity) are not achievable by current numerical simulations. However, the known heat flow and likely fluid motions for a dynamo suggest that tesseral harmonics of order 10^−8^ (corresponding to fractional density anomalies of this order or larger) might be present. This source is not pursued here because the observed anomaly changes from encounter to encounter in a way that resists a low-order static interpretation (see Supplementary Information).

Our analysis of Juno data shows that the retrieved p-modes amplitudes are compatible with those observed from the ground^9^. Fundamental (f-) modes are found to be small: their amplitudes are smaller than 1 cm/s, approximately 30–100 times smaller than p-modes, a ratio close to that observed on the Sun spectrum^21^. Our results indicate a peak radial velocity, at Jupiter’s radius, in the range 10–50 cm/s and that most of the power is in the range 900–1200 μHz. The Full Width at Half Maximum (FWHM, approx. equal to 0.5 *σ*_*f*_) is ~1/6–1/8 of the frequency peak, close to that of the Sun (~1/10 of the frequency peak).

At the present time, it is unknown if the similarities of Jupiter power spectrum to the Sun’s spectrum are purely accidental or if something more profound, like a common excitation mechanism, is at play. The frequency at which the maximum velocity is found can be related to the excitation source and may be indicative of the timescale at which energy is injected in the system. It would be very surprising if the Sun and giant planets behaved similarly because the luminosity of Jupiter is nine orders of magnitude smaller than for the Sun. Moreover, the actual density in the Sun is lower in the outermost region. As a consequence, the convective motions in the Sun are far stronger (and the fractional pressure fluctuations much larger) than those in Jupiter. In order for giant planets to exhibit large normal mode amplitude, the system has to be both highly non-dissipative (i.e., a high Q in the language often employed to describe tides) and avoid substantial mode-mode interaction that would cause a cascade of energy to other modes (of which there are a huge number). A highly non-dissipative environment is also required to sustain the strong zonal winds observed in giant planets. The existence of stronger winds on Saturn (compared to Jupiter) might be a clue to the stronger normal mode excitation in that planet and suggests no analogy to stars. The excitation and dissipation of modes in these planets is still not understood. A discussion on possible energy sources can be found in Dederick et al.^23^ and Markham et al.^24^.

Notwithstanding this puzzle, the detection of normal modes inside Jupiter and Saturn is a crucial step for the future exploration of the interior structure of gas giants, effectively paving the way to the discipline of seismology applied to this class of bodies. Unlike the detection reported here, future work can make use of the precise determination of mode frequencies and can detect modes at higher angular order (something not possible by Gaulme et al.^9^). Seismology can avoid much of the intrinsic ambiguity of gravity observations, even when (as with Juno) they are done to very high precision. Issues such as the nature of the core and the presence of regions of static stability can be discerned with seismology but are elusive with gravity. On the Sun, helioseismology has shown that the internal rotation profile presents a sharp separation into a rigidly rotating core and differentially rotating envelope. On Saturn, ring seismology proved that the interior is stably stratified by compositional gradients, with the core-envelope transition region extending to 60% of the planet’s radius^15^. Although powerful, ring seismology is very limited and not possible for most planets.

Our analysis of gravity data from Juno shows a new methodology for determining the normal modes amplitude spectra, which can be applied also to other solar system bodies^25^. The measurement of gravity perturbations produced by normal modes can provide information complementary with that of ground observations (future observations by, e.g., Schmider et al.^26^, Shaw et al.^27^), that is, the weak and low-frequency fundamental modes can be likely observed only through the gravitational perturbations they produce. If the orbit of a future mission can be tailored to perform accurate measurements of the time-variable component of the gravity field of a planet, it can provide exquisitely accurate information on the normal modes, both frequency and amplitudes. Furthermore, our analysis supports the presence of normal modes that can be much more accurately detected by dedicated instrumentation onboard future missions to the gas giant. The results that have been recorded here offer some insights into the design and range for a potential flight instrumentation, which can provide even more exciting results: it can be a powerful tool to probe the interior of gas and icy giants, similarly to what helioseismology has done for the Sun.

## Methods

### Data set

The current analysis extends the data set used in Durante et al.^4^ to also include Juno’s gravity-dedicated passes up to PJ33, for a total of 22 passes and 12299 Doppler points (at an integration time of 60 s). In a typical pass, the ground station tracks the spacecraft in a dual link (X- and Ka-band, 7.2–8.4 GHz and 34–32 GHz) two-way mode for 6–8 h around perijove; the outbound pass, whose primary scope is to monitor an orbit trimming maneuver (OTM), is collected by a different station. The data set used in the analysis includes Doppler data from the pericenter pass and the outbound pass, until when the OTM maneuver is executed. In most of the passes, the links are established by DSS-25 antenna of NASA’s Deep Space Network, located in Goldstone, California, in a X-up/X-down and Ka-up/Ka-down configuration. Combining X and Ka-band data allows removal of up to 75% of the noise due to charged particles. Only in a few passes (PJ01, PJ13, PJ27, PJ33) the Ka-band uplink signal was not available, and a X-up/X-down/Ka-down configuration was used, which only allow plasma calibration on the downlink leg. The dual-link configuration is extremely important due to the presence of the Io Plasma Torus, which introduces a delay and a Doppler shift in the data, thus potentially biasing the gravity estimation, if left uncalibrated. Moreover, the multi-link configuration reduces the total noise on Juno’s Doppler data by 10% on average.

The open-loop Doppler data have been compressed to 60 s, allowing sufficient sampling of the gravity signal of interest. The data have been calibrated for wet tropospheric noise with the Advanced Water Vapor Radiometer (AWVR), when available. AWVR data help in reducing the noise caused by fluctuations of water vapor content along the line-of-sight. We found a 34% reduction of the data noise on average (at an integration time of 60 s), with peaks of 65%, depending on the weather condition at (and close to) the ground station.

### Juno’s baseline dynamical model

The Doppler data have been analyzed with JPL’s MONTE orbit determination code^28^. The dynamical model of Juno’s orbit accounts for (see also Folkner et al.^29^, Iess et al.^2^, Durante et al.^4^): the gravitational accelerations of solar system planets, Jupiter and its satellites, in a relativistic 1-PN (post-Newtonian) formulation; the gravitational tides raised by the Sun and Galilean satellites on Jupiter; the motion of Jupiter’s spin axis in the plane of the sky; the non-gravitational accelerations caused by solar radiation pressure, Jupiter’s albedo and infrared emission, and the anisotropic thermal acceleration caused by solar panels difference in temperature.

The gravity field of Jupiter is modeled via zonal harmonics (even and odd, up to degree 30) as expected for a fluid body in rapid, differential, rotation. A full degree-2 tesseral field is included to look for possible deviation of the polar axis of inertia from the spin axis of Jupiter and equatorial ellipticity. We also account for the presence of the GRS with a dipole model for the mass anomaly^8^.

Concerning the gravitational response of Jupiter to its satellites, we have assumed it to be the same for all moons. Although Juno has in principle the capability to discriminate satellite-dependent tides^30^, the effect on Juno’s trajectory is smaller than that from the unknown accelerations we are focusing on. We included only Love numbers *k*_*lm*_ up to degree and order 4 having even *l-m*, since those with odd *l-m* are not observable due to the small inclination of the Galilean satellites.

The motion of Jupiter’s spin axis has been numerically integrated starting from the latest model from the international astronomical union (IAU)^31^, accounting for the gravitational torques of the Sun, the Galilean satellites acting on the planet assumed as a rigid body. The numerical integration is in agreement with the IAU model when the same initial conditions are used. The free parameters of the integration are the pole position at a given epoch and the polar moment of inertia factor.

Non-gravitational accelerations have been modeled similarly to Durante et al.^4^, with the exception of solar radiation pressure acceleration, whose model parameters have been updated after the analysis of Juno navigation data given by Notaro et al.^32^. The anisotropic thermal acceleration caused by the different temperature of the front and back sides of the solar array caused by Jupiter’s albedo and IR emission has been account for, although, being at most of the order of 8 × 10^−10^ m/s^2^ (i.e., ten times smaller than the solar radiation pressure), is too weak to contribute to the empirical accelerations (which are of the order of, at least, 2 × 10^−8^ m/s^2^).

The light-time computation used to generate Doppler observables is performed in a relativistic 1-PN framework, accounting also for the oblateness of Jupiter. Furthermore, we account for the Doppler shift induced by the bending of solar arrays have been included as well^33^ and for the newly estimated ground station delays^34,35^.

### Computing the number of degrees of freedom

In a multiarc least square estimation filter with *m* observables and *n* parameters, the correction to the state vector at each iteration, $\hat{{{{{{{{\boldsymbol{x}}}}}}}}}$, is:

11 $$\hat{{{{{{{{\boldsymbol{x}}}}}}}}}={\left({{{{{{{{\boldsymbol{H}}}}}}}}}^{{{{{{{{\boldsymbol{T}}}}}}}}}{{{{{{{\boldsymbol{WH}}}}}}}}+{\bar{{{{{{{{\boldsymbol{P}}}}}}}}}}^{-1}\right)}^{-1}{{{{{{{{\boldsymbol{H}}}}}}}}}^{{{{{{{{\boldsymbol{T}}}}}}}}}{{{{{{{\boldsymbol{Wy}}}}}}}}$$

where ***H*** is the matrix of partial derivatives of the observables with respect to state parameters, ***W*** is the observables weighting matrix, $\bar{{{{{{{{\boldsymbol{P}}}}}}}}}$ is the a priori covariance matrix of the state parameters, and ***y*** is the vector of observations residuals (observed minus computed values). $\bar{{{{{{{{\boldsymbol{P}}}}}}}}}$ contains a priori information only for normal mode amplitudes.

To compute the degrees of freedom of a given solution, we recall the relation, valid for a linearized model, between the observable quantities, ***y***, and the state parameters, ***x***:

12 $${{{{{{{\boldsymbol{y}}}}}}}}={{{{{{{\boldsymbol{Hx}}}}}}}}+{{{{{{{\boldsymbol{\epsilon }}}}}}}}$$

13 $$\hat{{{{{{{{\boldsymbol{y}}}}}}}}}={{{{{{{{\boldsymbol{H}}}}}}}}\left({{{{{{{{\boldsymbol{H}}}}}}}}}^{{{{{{{{\boldsymbol{T}}}}}}}}}{{{{{{{\boldsymbol{WH}}}}}}}}+{\bar{{{{{{{{\boldsymbol{P}}}}}}}}}}^{-1}\right)}^{-1}{{{{{{{{\boldsymbol{H}}}}}}}}}^{{{{{{{{\boldsymbol{T}}}}}}}}}{{{{{{{\boldsymbol{Wy}}}}}}}}=\hat{{{{{{{{\boldsymbol{H}}}}}}}}}{{{{{{{\boldsymbol{y}}}}}}}}$$

The trace of the $\hat{{{{{{{{\boldsymbol{H}}}}}}}}}$ matrix is the number of regression-effective degrees of freedom of a given solution^36^. Note that it does not depend on the observations, but only on ${{{{{{{\boldsymbol{H}}}}}}}}$ (which depends on the list of estimated parameters, including the normal mode amplitudes) and the a priori information matrix $\bar{{{{{{{{\boldsymbol{P}}}}}}}}}$ (which, again, depends on the normal modes parameters). Note that when the a priori information matrix is omitted (or contributes very little to the solution), $\hat{{{{{{{{\boldsymbol{H}}}}}}}}}$ is an identity matrix and the number of degrees of freedom reduces to the number of parameters.

## Supplementary information

Supplementary Information

## Data Availability

The raw tracking data and calibration files used in the analysis are available through NASA’s Planetary Data System^37^. The geometry of the Juno orbit, including SPK trajectory files and CK spacecraft attitude files, is available at https://naif.jpl.nasa.gov/pub/naif/JUNO/kernels/. The datasets generated during the current study are available from the corresponding author on reasonable request. Source data are provided with this paper.

## Source data

Source data

## Code availability

Distribution of the MONTE navigation code is restricted by the Export Administration Regulations of the US Department of Commerce. Eligible readers may request a copy of MONTE, under a license that does not permit redistribution, at https://montepy.jpl.nasa.gov/. The GYRE software is accessible from the repository https://github.com/rhdtownsend/gyre.
