# Supplementary material for: Juno spacecraft gravity measurements provide evidence for normal modes of Jupiter
Source: Nat Commun. 2022 Aug 30;13:4632. doi: 10.1038/s41467-022-32299-9 (PMC9427753; doi:10.1038/s41467-022-32299-9)
Supplement: Supplementary file 1 — Supplementary Information [file 41467_2022_32299_MOESM1_ESM.pdf]

Supplementary information:

Juno spacecraft gravity measurements provide evidence for normal modes of Jupiter

D. Durante, *et al.*

Corresponding author e-mail: daniele.durante@uniroma1.it

Alternative explanations

Normal modes are not the only possible solution that can be invoked to fit Juno's Doppler data. As we anticipated, other possibilities include non-zonal atmospheric dynamics, or density anomalies at depth, possibly related to the interaction between the atmosphere and the magnetic field. To support normal modes detection with Juno's data, we investigated if other estimation approaches, based on physical assumptions, can provide solutions as likely as those from normal modes.

First, we start from a model using empirical accelerations, not related to any physical phenomenon, to understand the characteristics of the unmodelled accelerations. Then, we investigate a static tesseral field or localized density anomalies, possibly related to a non-zonal atmospheric dynamic or deeply seated density anomalies in the dynamo region.

Empirical accelerations

The approach of using empirical piecewise-constant accelerations to account for unknown accelerations in Juno's dynamical model has been successfully used by Durante, et al.[1] in the data analysis of Juno's data collected until halfway into the nominal mission (10 passes). Similarly, Iess, et al.[2] took advantage of empirical accelerations to properly fit Cassini's data during the Grand Finale phase of the mission, when Cassini passed very close to Saturn in a Juno-like orbit.

The empirical accelerations model tries to reproduce unknown accelerations in the dynamical model by estimating piecewise-constant accelerations over a given timespan (± 1 hour about pericenter), constant in Juno's RTN frame (defined by the radial, transverse, normal directions in Juno's osculating orbit), with a time update of a few minutes (10 minutes in the previous analysis) and a constraining a priori uncertainty on the amplitudes. Thus, the relevant parameters of the model are the time update and the a priori uncertainty. As a general rule, the parameters of the empirical accelerations model can be adjusted to obtain a proper fit of the data, with a tradeoff between model complexity and goodness of fit.

If we use the very same set of empirical accelerations parameters of Durante, et al.[1] for analyzing the larger dataset used in the current work, the fit of the Doppler data is not satisfactory, as residual signature close to the pericenter are quite apparent in a few passes (see columns two and five of Supplementary Figure 1). Therefore, we tweaked the model parameters to find more suitable solutions.

Supplementary Figure 2 reports the ΔAIC value as a function of the model parameters while keeping the timespan fixed to ± 1 hour about pericenter (a larger timespan would not improve the solution since Juno is very far from Jupiter and the perturbing accelerations would be small). ΔAIC is defined using the classical definition (no contribution from normal modes RSS), whilst the zero value corresponds to the best normal mode solution found. Recall that Durante, et al.[1] used a time update of 10 minutes and an a priori uncertainty of 2×10^{-8} m/s^2 (a combination that worked since the dataset was limited). The results indicate that to obtain a solution with AIC values as good as the best ones using normal modes, short time updates of 3–4 minutes and an a priori uncertainty of at least 5×10^{-8} m/s^2 are required (in 3 min Juno travels approximately 11000 km, or 9° in latitude). This is an indication that the accelerations the model attempts to mimic have a high frequency.

As a comparison, the most likely normal modes solution has degree 2 harmonic coefficients as large as 10^{-8}, which translate to a peak acceleration of about 6×10^{-7} m/s^2 at Juno's pericenter, with a characteristic period of ~15 minutes. Thus, in order to produce comparable accelerations, it is not surprising that the empirical model converges to a shorter time span and larger magnitude than the one previously used. Phenomena that have a typical time scale of 15 minutes cannot be reproduced with a time update of 10 minutes.

Static tesseral field

Giant planets are rapid rotators, meaning that the rotational, mostly hydrostatic distortion of the planet is not small. However, the Rossby number $Ro \equiv v/2\Omega L \ll 1$, i.e., the non-rotational fluid motions are small (v is fluid velocity, Ω is the rotation rate, L is the characteristic length scale). Moreover, they are low viscosity planets, meaning that fluid convection can easily carry the heat flow with only very small temperature and density anomalies. As a consequence, the gravitational field is dominated by the non-spherical but spin-axisymmetric distortion of equipotential surfaces expressed in a zonal gravity field. However, these planets do convect and have magnetic fields; these break the spin-axisymmetry and allow for tesseral harmonics (irrespective of the possible presence of normal mode excitation, which should also break the symmetry). No reliable models exist for this possibility, but their possible amplitude can be estimated by considering the density fluctuations that arise from a specified (but likely) velocity field. Deep down (where the density is high so small density fluctuations can still be a good source of gravity fluctuations), observations of possible magnetic field variation[3] suggest low velocities of order 1 cm/s or less; we use that here as a scale for consideration. First, consider the equation of motion: in rapidly rotating low viscosity fluids, much of the Coriolis force is plausibly balanced by gradients in dynamical pressure:

$$\delta p \sim 2\rho\Omega v L \tag{1}$$

where L is some large lateral length scale. Since pressure fluctuation implies density fluctuation through the compressibility of hydrogen, we obtain the estimated fractional density anomalies:

$$\frac{\delta\rho}{\rho} \sim 10^{-8}\left(\frac{v}{1\ cm\ s^{-1}}\right) \tag{2}$$

to order of magnitude. We can alternatively consider the heat flux F as a guide. Using the formula:

$$F = \rho C_p < v\delta T > \tag{3}$$

for convective heat flux, where v and δT are now vertical velocity and temperature anomalies, and relating the temperature anomaly to density anomaly using the Maxwell relations and Grunëisen gamma ~1, we obtain:

$$\frac{\delta\rho}{\rho} \sim \frac{F}{\rho c^2 v} \sim 10^{-8}\left(\frac{v}{1\ cm\ s^{-1}}\right) \tag{4}$$

where c is the speed of sound. These two estimates are roughly comparable but with very different physical considerations. If the density anomaly occurs in a region where the density is similar to the mean density of Jupiter and has a large associated length scale, then the resulting gravity anomaly C_{lm} is of similar magnitude (10^{-8}). Of course, it can easily be smaller and the velocity anomalies are not known so this is only intended to show that it is plausible to have tesserals of the same order as those that might be needed to explain the data.

Supplementary Figure 3 reports the ΔAIC value of solutions as a function of the maximum degree and order of the tesseral field expansion, for different a priori uncertainties imposed on the tesseral coefficients. The blue and orange lines refer to solutions in which the RMS of tesseral coefficients have been constrained to thermal wind model predictions[4,5] of a non-zonal flow decaying to 500 km or 1000 km. The first case agrees with findings of the depth of the GRS[6], while the second case depicts stronger, i.e., deeper, non-zonal winds. Our results indicate that even a large degree and order field constrained by reasonable assumptions in term of plausible atmospheric dynamic perturbations does not fit the data as well as solutions with normal modes. Moreover, the reconstructed fields show high order tesseral coefficients with amplitudes much larger than the expectations from surface winds. That is, the data favors very local anomalies, or short spatial scales. The green and violet lines refer, respectively, to solutions having a priori uncertainties roughly based on density

anomalies predictions in the dynamo region[7], for an RMS flow of 1 cm/s or 10 cm/s. Good solutions are obtained only in the case of a large degree and order field (larger than 16), with strong flows in the dynamo region. However, such a flow would produce a correction to J_3 which is not compatible with models of the deep atmospheric dynamic and zonal winds strength. We conclude that such solutions appear to be physically implausible.

Localized density anomalies

An alternative approach which may be used to fit Juno's data is based on the concepts of mascons, i.e., localized density anomalies. A similar approach has been successfully used to constrain the depth of Jupiter's GRS[6], with the density anomalies being modelled as a dipolar structure. Such model is motivated by thermal wind balance, which predicts a top mass anomaly having equal magnitude but opposite sign with respect to the bottom mass anomaly.

In our analysis, we place point mascons, treated as local parameters, along each of Juno's ground tracks with a fixed latitudinal spacing and explore the parameter space given by the a priori uncertainty imposed on the mass anomalies by the estimation filter. Recall that the GRS spans about 10° in latitude. To compare our localized masses with that of the GRS, we must convert between the two models (dipole structure and a single point mascon). The estimated mass for the GRS dipolar structure is GM ~0.1 km^3/s^2, which produces an acceleration on Juno of about $3x10^{-8}$ m/s^2 at an altitude of 13000 km (reached during the PJ18 overflight of the GRS), or $5x10^{-7}$ m/s^2 at a typical altitude of Juno's pericenter. To experience the same acceleration with a single mascon, we require a smaller mass: GM ~0.005 km^3/s^2 at Juno's PJ18 altitude (13000 km) or GM ~0.01 km^3/s^2 at Juno's pericenter altitude (about 5000 km). This is a factor 10 to 20 smaller than the masses involved in the dipolar mascon model.

Supplementary Figure 4 reports the ΔAIC value for the different solutions, for a few latitudinal spacing. Our analysis indicates that mass anomalies larger than 0.03 km^3/s^2 (with a latitudinal spacing of 2.5° to 5°) or larger than 0.1 km^3/s^2 (with a spacing of 10°, comparable to GRS dimension) would provide solutions with ΔAIC values as low as those obtained with normal modes. However, such solutions require large mass anomalies randomly distributed on Jupiter: 10 times larger than those involved in the GRS, which is by far the largest atmospheric feature on Jupiter.

The GRS is a shallow phenomenon, with a depth of about 500 km. One cannot exclude the presence of localized mass anomalies caused by atmospheric dynamics well below the cloud level, possibly at depth. However, the cause and persistence of such strong density anomalies is, at the moment, unexplained.

Supplementary Table 1 | Summary of orbital geometry and tracking configuration, for all perijove (PJ) passes. Latitude and longitude coordinates are given in System III[8]; altitude is with respect to the oblate Jupiter. The angle between the negative orbit normal (NON) and Earth's direction determines the projection of Juno's velocity along the line-of-sight. The Sun-Earth-probe (SEP) angle controls the plasma noise contribution to the radio links. The root-mean-square (RMS) of the open-loop Doppler points, 60 s integration time, converted to two-way range-rate, is given for the perijove tracking pass only.

PJs	Date (UTC)	Latitude & longitude	Altitude (km)	NON-to-Earth (°)	SEP (°)	Doppler points at 60 s & tracking configuration	RMS (mm/s)
PJ01	27 Aug. 2016 12:50:44	3.8°, 264.2°	4162.8	2.8	22.6	457 (X/X+X/Ka, DSS 55)	0.106
PJ03	11 Dec. 2016 17:03:41	5.6°, 354.5°	4153.2	19.2	61.4	383 (X/X+Ka/Ka, DSS 25) + 275 (X/X, DSS 43)	0.018
PJ06	19 May 2017 06:00:43	8.7°, 220.2°	3501.6	15.1	135.5	457 (X/X+Ka/Ka, DSS 25) + 244 (X/X, DSS 43)	0.014
PJ08	1 Sep. 2017 21:48:50	10.4°, 41.0°	3500.5	23.4	42.7	417 (X/X+Ka/Ka, DSS 25) + 87 (X/X, DSS 43)	0.016
PJ10	16 Dec. 2017 17:57:38	10.5°, 64.1°	4276.9	45.5	40.7	360 (X/X+Ka/Ka, DSS 25) + 247 (X/X, DSS 43)	0.019
PJ11	7 Feb. 2019 13:51:49	12.2°, 154.6°	3468.9	53.2	86.8	361 (X/X+Ka/Ka, DSS 25) + 120 (X/X, DSS 36)	0.011
PJ13	24 May 2018 05:39:50	14.8°, 335.9°	3497.9	48.2	163.4	391 (X/X+X/Ka, DSS 26) + 159 (X/X, DSS 43)	0.024
PJ14	16 Jul. 2018 05:17:22	15.7°, 291.5°	3498.0	45.3	109.7	385 (X/X+Ka/Ka, DSS 25) + 357 (X/X, DSS 43)	0.018
PJ15	7 Sep. 2018 01:11:40	16.6°, 22.0°	3499.2	49.8	63.6	334 (X/X+Ka/Ka, DSS 25) + 323 (X/X, DSS 43)	0.030
PJ17	21 Dec. 2018 16:59:48	18.1°, 203.4°	5053.3	70.8	20.2	43 (X/X, DSS 55) + 309 (X/X+Ka/Ka, DSS 25) + 127 (X/X, DSS 43)	0.026
PJ18	12 Feb. 2019 17:34:31	18.9°, 124.7°	3499.4	80.6	64.0	309 (X/X+Ka/Ka, DSS 25) + 257 (X/X, DSS 43)	0.014
PJ21	21 Jul. 2019 04:02:43	21.0°, 81.4°	7975.7	75.7	137.1	280 (X/X, DSS 55) + 330 (X/X+Ka/Ka, DSS 25) + 132 (X/X, DSS 43)	0.012
PJ22	12 Sep. 2019 03:40:44	21.7°, 36.7°	7974.2	76.2	86.9	32 (X/X, DSS 54) + 317 (X/X+Ka/Ka, DSS 25) + 353 (X/X, DSS 43)	0.011
PJ23	03 Nov. 2019 22:18:14	22.5°, 173.8°	3500.5	85.9	42.7	298 (X/X+Ka/Ka, DSS 25) + 67 (X/X, DSS 43)	0.010
PJ25	17 Feb. 2020 17:51:55	23.5°, 219.4°	4700.9	107.1	41.3	304 (X/X+Ka/Ka, DSS 25) + 230 (X/X, DSS 35)	0.011
PJ27	02 Jun. 2020 10:18:38	24.7°, 17.1°	3498.9	114.9	135.7	344 (X/X+X/Ka, DSS 25) + 144 (X/X, DSS 36)	0.018
PJ28	25 Jul. 2020 06:15:27	25.3°, 107.2°	3500.3	108.8	168.2	216 (X/X, DSS 55) + 337 (X/X+Ka/Ka, DSS 25) + 155 (X/X, DSS 34)	0.016
PJ29	16 Sep. 2020 02:10:52	25.9°, 197.3°	3499.6	104.7	113.7	44 (X/X+X/Ka, DSS 54) + 312 (X/X+Ka/Ka, DSS 25) + 132 (X/X, DSS 35)	0.011
PJ30	08 Nov. 2020 01:49:42	26.6°, 152.3°	3498.9	108.5	66.0	218 (X/X, DSS 25) + 89 (X/X+Ka/Ka, DSS 25) + 290 (X/X, DSS 34)	0.037
PJ31	30 Dec. 2020 21:45:44	27.3°, 242.3°	3499.1	117.8	22.9	324 (X/X+Ka/Ka, DSS 25)	0.017
PJ32	21 Feb. 2021 17:40:34	28.0°, 332.3°	4883.1	129.0	18.4	316 (X/X+X/Ka, DSS 55) + 352 (X/X+Ka/Ka, DSS 25) + 73 (X/X, DSS 43)	0.022
PJ33	15 Apr. 2021 23:32:25	28.8°, 62.0°	3499.3	138.8	59.9	556 (X/X+X/Ka, DSS 34)	0.047

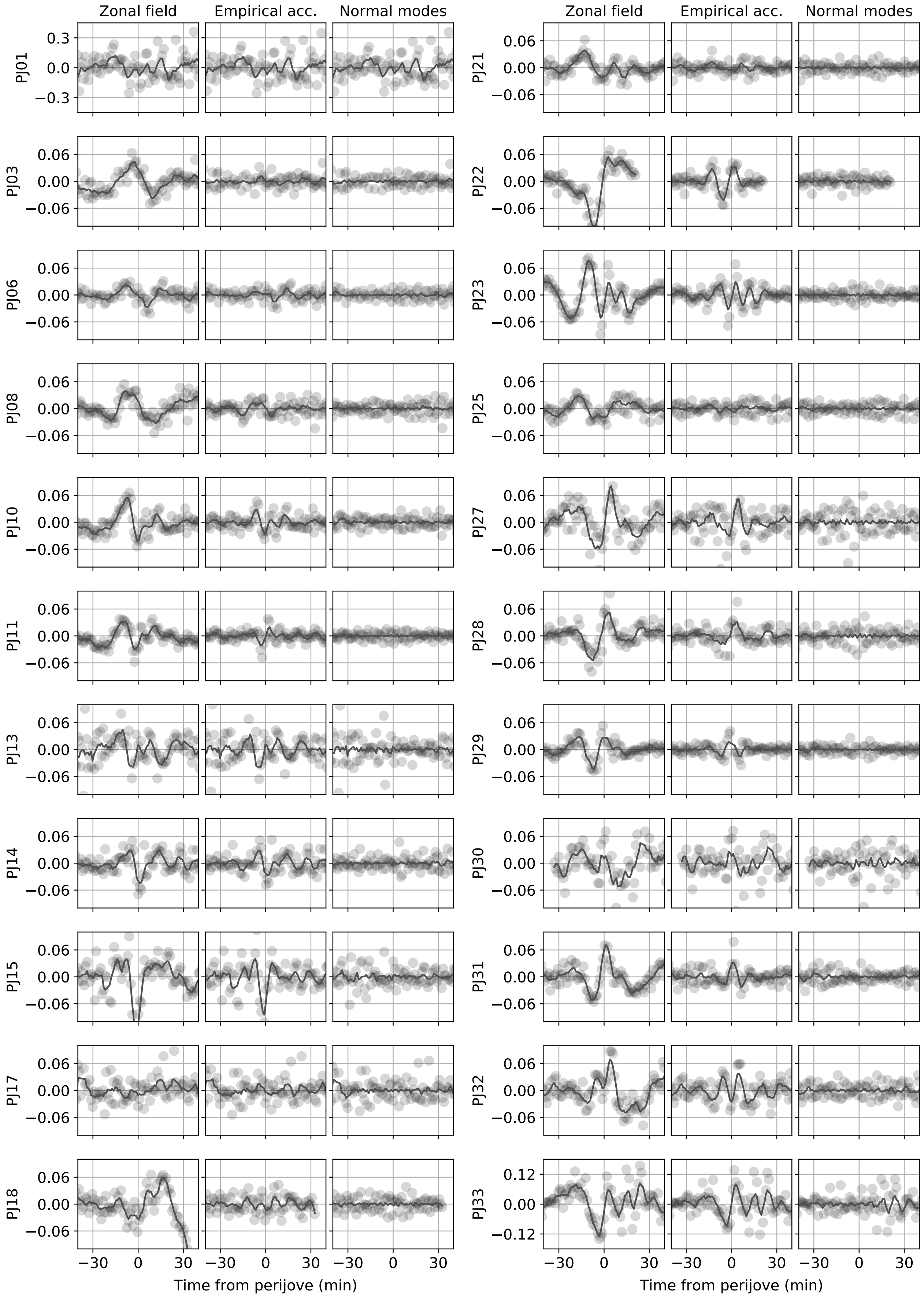

Supplementary Figure 1 | Juno two-way range-rate (Doppler) residuals in mm/s for all Juno perijove passes and different dynamical models. First (and fourth) column: static zonal gravity field; second (and fifth) column: empirical accelerations at the level of Durante, et al.[1]; third (and last) column: best normal modes solution

found in the analysis. The light black line is a moving average of the residuals highlighting signatures near perijove. The inclusion of empirical accelerations reduces the signatures in the data, when compared with the solution obtained assuming a static zonal gravity field; the best fit is obtained with normal modes (Doppler residuals are consistent with white-frequency noise).

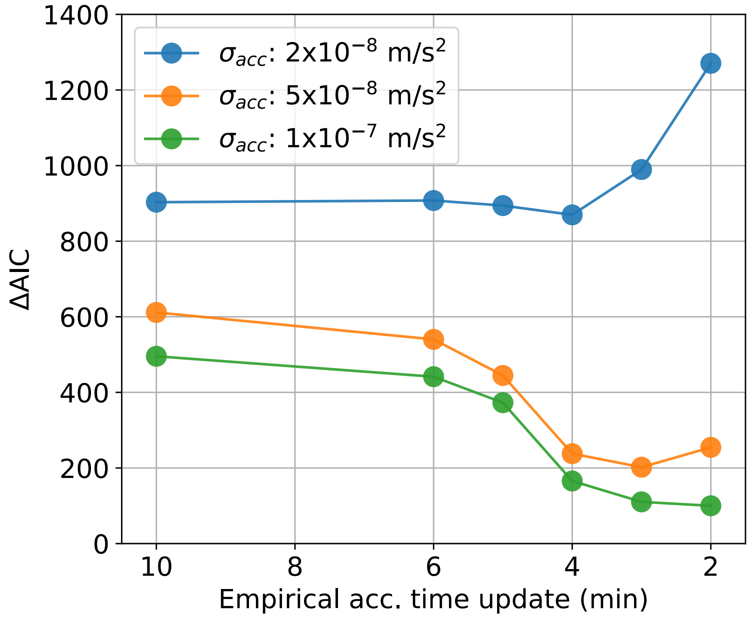

Supplementary Figure 2 | ΔAIC value of solutions including empirical accelerations as a function of the time update interval. The curves are for different levels of the a priori uncertainty σ_{acc} on the accelerations.

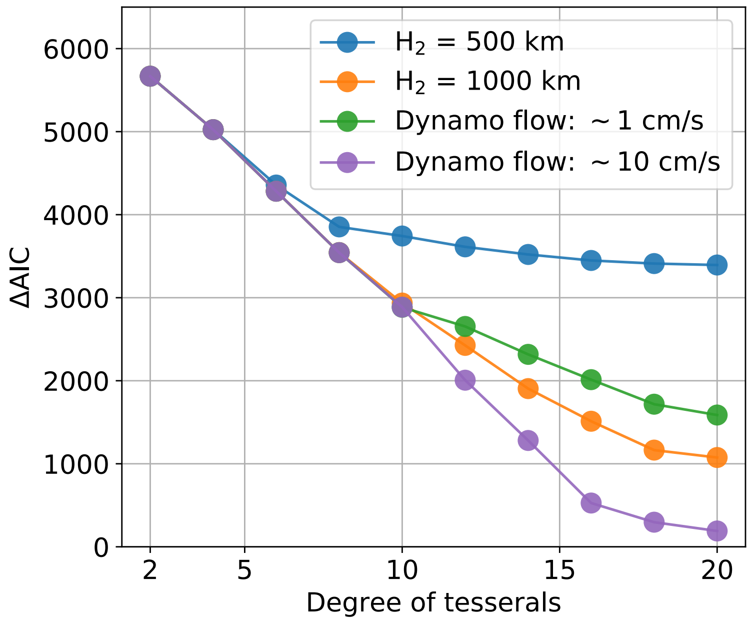

Supplementary Figure 3 | ΔAIC value as a function of the maximum degree and order for the expansion of the static gravity field. Curves are for different sets of a priori uncertainties (H_2 is the decay depth of the non-zonal flow). The field is fixed to System III reference frame[8].

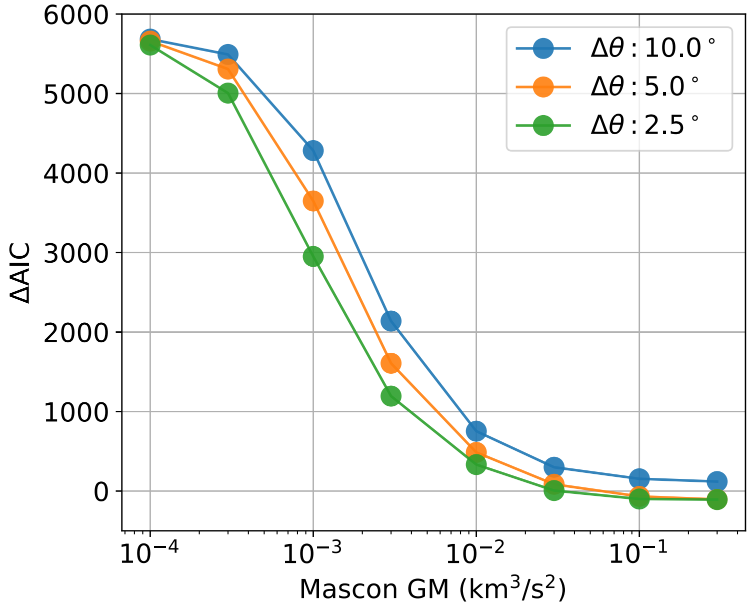

Supplementary Figure 4 | ΔAIC value as a function of the mass anomaly hidden underneath Juno's ground tracks. Curves are for different latitudinal $\Delta\theta$ spacing between the mascons. For comparison, the GRS has an equivalent mascon GM[6] of 0.005–0.01 km^3/s^2, with a spatial dimension of about 10°.

Supplementary references

1. Durante, D., *et al.* Jupiter's gravity field halfway through the Juno mission. *Geophysical Research Letters* **47**, 4 (2020)
2. Iess, L., *et al.* Measurement and implications of Saturn's gravity field and ring mass, *Science* **364**, aat2965 (2019)
3. Moore, K.M., *et al*. Time variation of Jupiter's internal magnetic field consistent with zonal wind advection. *Nature Astronomy* **3**, 8, 730–735 (2019)
4. Parisi, M., Galanti, E., Finocchiaro, S., Iess, L. & Kaspi, Y. Probing the depth of Jupiter's Great Red Spot with the Juno gravity experiment. *Icarus* **267**, 232–242 (2016).
5. Galanti, E., Durante, D., Finocchiaro, S., Iess, L. & Kaspi, Y. Estimating Jupiter's gravity field using Juno measurements, trajectory estimation analysis, and a flow model optimization. *Astronomical Journal* **154**, 1 (2017).
6. Parisi, M., *et al*. The depth of Jupiter's Great Red Spot constrained by the Juno gravity overflights. *Science* **374**, 964–968 (2021).
7. Kulowski, L., Cao, H. & Bloxham, J. Contributions to Jupiter's Gravity Field from Dynamics in the Dynamo Region. *Journal of Geophysical Research: Planets* **125** (2020)
8. Seidelmann, P.K., & Divine, N. Evaluation of Jupiter longitudes in System III (1965). *Geophysical Research Letters* **4**, 65–68 (1977).
